# Integrative Survival Prediction in Breast Cancer Using Extracellular Matrix Protease Transcript Signatures and Clinical Variables: A Machine Learning Approach

**DOI:** 10.3390/cancers18101497

**Published:** 2026-05-07

**Authors:** Rami Babas, Demitrios H. Vynios, Aristotelis Kompothrekas, Basilis Boutsinas, Nikos Karamanos

**Affiliations:** 1 Biochemistry, Biochemical Analysis & Matrix Pathobiochemistry Research Group, Department of Chemistry, University of Patras, 26504 Patras, Greece; up1118904@upatras.gr; 2 Management Information Systems and Business Intelligence Laboratory, University of Patras, 26504 Patras, Greece

**Keywords:** breast cancer, prognostic biomarkers, MMPs, ADAM, ADAMTS, integrative survival modeling, molecular subtypes, precision oncology, machine learning

## Abstract

**Background/Objectives**: Traditional breast cancer prognostic tools relying on clinical staging often miss molecular heterogeneity, leading to divergent patient outcomes. Extracellular matrix (ECM) remodeling, driven by the Matrix Metalloproteinase (MMP), ADAM, and ADAMTS enzyme families, is critical to tumor progression. This study evaluates whether integrating ECM protease transcript abundance with standard clinical variables improves survival prediction accuracy and personalized risk stratification. **Methods**: Clinical and transcriptomic data from The Cancer Genome Atlas (TCGA) breast cancer cohort were analyzed. We integrated the protein-coding transcripts per million (pTPM) of top-ranked protease genes with standard clinical covariates (age, ordinal stage). Cox Proportional Hazards (CoxPH), penalized Cox (CoxNet), Random Survival Forest (RSF), and Gradient Boosting Survival (GBS) models were evaluated under a stratified 70/30 train–test split, followed by five-fold cross-validation. The locked final RSF model was then externally tested in METABRIC without retraining or risk-cutoff optimization. **Results**: Univariate screening identified ADAM15, MMP15, and ADAMTSL1 as global risk factors, whereas ADAMTS8 and MMP7 were protective. Prognostic signals were subtype-dependent. Integrated multivariable models outperformed transcript-only approaches in internal testing. The integrative RSF achieved the highest held-out discrimination (C-index = 0.797), outperforming a clinical-only Cox baseline trained on age and stage alone (C-index = 0.742, 95% CI 0.636–0.826). In METABRIC, the external C-index was 0.581 (95% CI 0.562–0.598), with significant survival separation across training-defined risk groups (log-rank *p* < 0.0001). **Conclusions**: ECM protease transcript profiles provide complementary prognostic information in TCGA-BRCA and show partial transportability to METABRIC. However, the modest external C-index indicates limited individual-level discrimination across platforms, so these candidate markers should be interpreted as hypothesis-generating and require further validation before clinical implementation.

## Simple Summary

Breast cancer is among the most common malignancies globally, with incidence and mortality placing it at the forefront of oncological research. Patients with similar clinical profiles can experience markedly different outcomes, largely because traditional staging tools fail to capture molecular heterogeneity. This study addresses that gap by integrating transcriptional abundance of extracellular matrix proteases, specifically the MMP, ADAM, and ADAMTS families, with standard clinical variables to improve survival prediction. The models successfully identified “hidden high-risk” patterns in TCGA-BRCA. Independent testing in METABRIC showed statistically significant risk-group separation but only modest individual-level discrimination (external C-index = 0.581), indicating that the signature is promising but requires further platform-harmonized and prospective validation before clinical use.

## 1. Introduction

Breast cancer remains a leading cause of cancer morbidity and mortality worldwide, with an estimated 2.3 million new cases and 685,000 deaths recorded globally in 2020 [1]. Accurate prognostic stratification is central to treatment planning and follow-up, and decision support has evolved from classical clinical indices such as the Nottingham Prognostic Index [2] and Adjuvant! Online [3] toward guideline-driven frameworks [4] and multigene genomic assays such as Oncotype DX [5]. Even with these advances, clinically similar patients can show different outcomes, and heterogeneity across tumor subtypes limits the precision of single-modality tools [6].

Molecular assays typically rely on transcriptomic signatures, yet post-transcriptional regulation can decouple mRNA from protein abundance and activity, motivating targeted feature sets that capture pathways linked to invasion and microenvironment remodeling [7]. Extracellular matrix (ECM) remodeling is a core component of tumor progression [8]. Matrix metalloproteinases (MMPs) are a family of zinc-dependent endopeptidases that degrade ECM components and regulate signaling through substrate processing [9]; their structure, function, and biochemical regulation have been extensively characterized [9,10]. A network view of protease activity frames ECM remodeling as a system of coordinated interactions rather than isolated enzyme effects [11]. The ADAM (A Disintegrin and Metalloproteinase) family contributes through ectodomain shedding and pathway modulation [12,13], while ADAMTS (A Disintegrin and Metalloproteinase with Thrombospondin Motifs) members regulate stromal interactions that influence invasion, immune interactions, and treatment response [14,15].

MMP expression has been associated with adverse outcomes across breast cancer subtypes [16,17]. MMP-1 holds independent prognostic value in breast cancer [18], MMP-9 drives malignant progression in basal-like triple-negative breast cancer [19], and MMP-11 has been shown to correlate inversely with CD8+ T cell infiltration and survival [20,21]. Subtype-specific MMP expression patterns further underscore the context-dependence of these effects [22]. Among ADAMs, ADAM8 promotes invasion and metastasis in breast cancer [23] and is broadly expressed with prognostic implications in hormone receptor-positive, HER2-negative disease [24]; inhibitory strategies against ADAM8 are under active investigation [25]. ADAM9 drives triple-negative breast cancer (TNBC) progression via the AKT/NF-κB pathway [26], ADAM10 modulates Notch1 signaling and CD44 expression [27,28], ADAM12 predicts outcome in estrogen receptor-positive breast cancer [29], and ADAM17 independently predicts adverse outcome [30]. Collectively, ADAMs contribute to TNBC via the mTORC1 pathway [31], and receptor ectodomain shedding represents a mechanistic axis for both treatment resistance and monitoring [13]. ADAMTS family members have been linked to genetic disorders and cancer biology [14,15], and candidate extracellular proteases more broadly represent a promising class of prognostic markers [32].

This study tests the hypothesis that ECM protease transcript abundance provides complementary prognostic information beyond standard clinical variables. To maximize both predictive power and clinical utility, continuous machine learning survival algorithms are applied to evaluate non-linear risk trajectories and generate personalized risk stratification.

## 2. Materials and Methods

### 2.1. Data Sources and Variables

Clinical data and tumor annotations were derived from The Cancer Genome Atlas (TCGA) breast cancer cohort [33], and transcriptomic data access and harmonization used UCSC Xena workflows [34]. GTEx resources were referenced for expression context in the source workflow [35].

The external validation dataset was obtained from the METABRIC cohort through cBioPortal/DataHub [36,37].

Clinical covariates used in downstream modeling were age at diagnosis (continuous) and clinical stage (ordinal, I–IV). PAM50-based molecular subtype was used for subtype-stratified screening only and not as a continuous modelling covariate. Race was not used in any model, as TCGA clinical annotation is racially imbalanced and a meaningful race-stratified analysis was not feasible in the subtype-stratified subsets.

The demographic and clinical characteristics of the study cohort are summarized in Table 1. The cohort comprised 942 patients with complete survival and expression data. Molecular subtype distribution was: Luminal A (*n* = 433, 46.0%), Basal-like (*n* = 155, 16.5%), Luminal B (*n* = 156, 16.6%), HER2-positive (*n* = 61, 6.5%), and Normal-like (*n* = 34, 3.6%), with the remaining patients classified as unknown subtype and excluded from subtype-specific analyses. Clinical stage distribution across the cohort was: Stage I (*n* = 170, 18.0%), Stage II (*n* = 529, 56.2%), Stage III (*n* = 212, 22.5%), Stage IV (*n* = 16, 1.7%), and missing/unparseable stage (*n* = 15, 1.6%). The TCGA BRCA samples were collected between approximately 1989 and 2013, spanning a period during which standard-of-care treatments evolved from anthracycline-based chemotherapy and tamoxifen toward aromatase inhibitors, taxane regimens, and HER2-targeted therapies (trastuzumab approved 1998).

### 2.2. Study Design

Overall survival was defined as time to death from any cause, with censoring at last follow-up for individuals alive at last contact. The workflow harmonized survival status into a binary event indicator and filtered records with missing or non-positive time.

#### 2.2.1. Preprocessing

Survival status was harmonized to a binary event indicator (1 = deceased; 0 = alive/censored). Age was coerced to numeric and imputed using the cohort median where missing. Stage strings were converted into an ordinal variable by extracting Roman numerals and mapping Stage I to 1, II to 2, III to 3, and IV to 4, with a value of 0 assigned for unparseable or missing entries. Protease expression features were coerced to numeric, zero-imputed where missing, and transformed with log1p to reduce right skewness. All protease features together with age, and the ordinal stage variable were then standardized by z-score scaling (mean = 0, SD = 1) applied to the full cohort. The fitted scaler was retained for consistent application at prediction time.

#### 2.2.2. Univariate Screening

Univariate Cox proportional hazards models (CoxPHFitter; lifelines library v0.27) were fitted independently for each protease feature to screen for association with overall survival. Features were ranked by ascending *p*-value, and the top 15 proteases were carried forward as the protein feature set for all subsequent modelling. Subtype-specific Cox screening repeated this process within each molecular subtype, excluding subtypes with fewer than 30 samples and records labelled as unknown subtype to reduce model instability Subtype-specific results for all proteins are presented in Table A1.

#### 2.2.3. Feature Sets for Modeling

Models compared:

- pTPM-only feature sets based on top-ranked proteases from univariate screening.
- Integrated clinical plus pTPM feature sets using age, stage ordinal, and the top-ranked protease features.

Four model families were evaluated under identical feature sets:

1. Cox proportional hazards (CoxPHSurvivalAnalysis).
2. Elastic net Cox (CoxnetSurvivalAnalysis, l1 ratio 0.5).
3. Random Survival Forest (RSF).
4. Gradient Boosting Survival (GBS).

For the held-out model comparison, RSF was configured with 100 trees, maximum depth 5, minimum samples per split 10, and minimum samples per leaf 5. GBS was configured with 100 estimators, maximum depth 3, and a learning rate of 0.10. CoxNet used an elastic-net mixing parameter (l1_ratio = 0.5) fitted over 100 alpha values on a regularisation path (alpha_min_ratio = 0.01, max_iter = 200,000). The final integrative RSF was subsequently re-trained on the full cohort using 200 trees, maximum depth 5, minimum samples per split 10, minimum samples per leaf 5, and square-root feature sub-sampling (max_features = ‘sqrt’).

#### 2.2.4. Internal Validation

A stratified 70%/30% train–test split (stratified by event status; random seed 1000) supported held-out model comparison. The primary discrimination metric was the concordance index (C-index), which measures the model’s ability to correctly rank pairs of patients by predicted risk. The final integrative RSF underwent five-fold cross-validation (shuffled KFold; random seed 1000) to estimate mean out-of-sample performance and fold-level variability. All analyses were implemented in Python (v3.10, Python Software Foundation, Wilmington, DE, USA). Survival models were built using scikit-survival v0.21 (Random Survival Forest, Gradient Boosting Survival Analysis, CoxPH Survival Analysis, and Coxnet Survival Analysis) and lifelines 0.27 (CoxPH Fitter, for univariate screening). Data manipulation relied on pandas 2.0 and numpy 1.24; preprocessing and cross-validation used scikit-learn 1.3. Visualizations were produced with matplotlib v3.7 (NumFOCUS, Austin, TX, USA) and seaborn v0.12 (a full comparison of model performance across all feature sets and model families is provided in Table A2).

The full analysis code is available from the corresponding authors (Appendix C). During the preparation of this study, the authors used Anthropic Claude Sonnet 4.6 (https://claude.ai, Anthropic PBC, San Francisco, CA, USA, accessed 2026) to assist in diagnosing and understanding code errors during analysis implementation. All code was written, reviewed, and validated by the authors, who take full responsibility for the final analysis.

#### 2.2.5. Statistical Validation of Risk Group Separation

Observed overall survival across the three RSF-derived risk tertiles (Low, Medium, High) was compared using a multivariate log-rank test (lifelines.statistics.multivariate_logrank_test) on the full cohort (*n* = 942). Pairwise Low vs. Medium, Low vs. High, and Medium vs. High contrasts were then computed and corrected for multiple testing using the Bonferroni method. Kaplan–Meier survival curves for the three groups, with 95% confidence-interval bands and per-group number-at-risk tables, were produced with lifelines. KaplanMeierFitter. All tests were two-sided with α = 0.05.

#### 2.2.6. Risk Stratification and Patient-Level Prediction

The final RSF model generated a continuous risk score for each patient (the ensemble-averaged cumulative hazard). Patients were stratified into Low, Medium, and High-risk groups by dividing the risk score distribution into equal tertiles. Patient-level survival curves were derived from the RSF survival function, and survival probabilities were extracted at fixed time horizons of 1, 2, 3, and 5 years. Permutation-based feature importance was computed by measuring the mean decrease in C-index when each feature was randomly permuted across 20 repetitions (random seed 1000).

#### 2.2.7. External Independent Validation

To satisfy the requirement for external independent validation, the final trained Random Survival Forest (RSF) model was locked after training exclusively on the TCGA-BRCA discovery cohort (*n* = 942 with complete survival and expression data). No retraining or parameter tuning was performed on the external data at any stage. The METABRIC cohort (Molecular Taxonomy of Breast Cancer International Consortium) was used as the independent external validation dataset. Clinical survival data were obtained from the METABRIC data release on cBioPortal, comprising 2509 patients, of whom 1979 had both Illumina microarray gene-expression profiles and complete overall survival data available after the time > 0 filter applied in the validation script. Overall survival status was encoded as a binary event (1 = deceased, 0 = living) and survival time was converted from months to days (×30.4375). Because METABRIC used Illumina microarray log2-intensity data whereas the discovery cohort used RNA-Seq pTPM values, direct expression values are not comparable across platforms. To avoid systematic bias, the same log1p transformation applied during training was first applied to METABRIC expression data, after which an independent z-score normalization was fitted and applied solely on METABRIC samples with no information from the training scaler. One gene nomenclature discrepancy was identified (MMP23 in training corresponds to MMP23B in METABRIC); this alias was mapped explicitly. Risk scores were predicted for all 1979 METABRIC patients using the locked RSF model. Risk group stratification (Low/Medium/High tertiles) used cutoff thresholds derived from the 33rd and 67th percentiles of the training cohort risk score distribution, applied blindly to METABRIC. External discriminative performance was quantified by the concordance index (C-index) with a 1000-iteration bootstrap 95% confidence interval. Risk group separation in METABRIC was assessed by Kaplan–Meier survival curves and a multivariate log-rank test.

## 3. Results

### 3.1. Full Cohort Survival Associations

**Figure 1:**
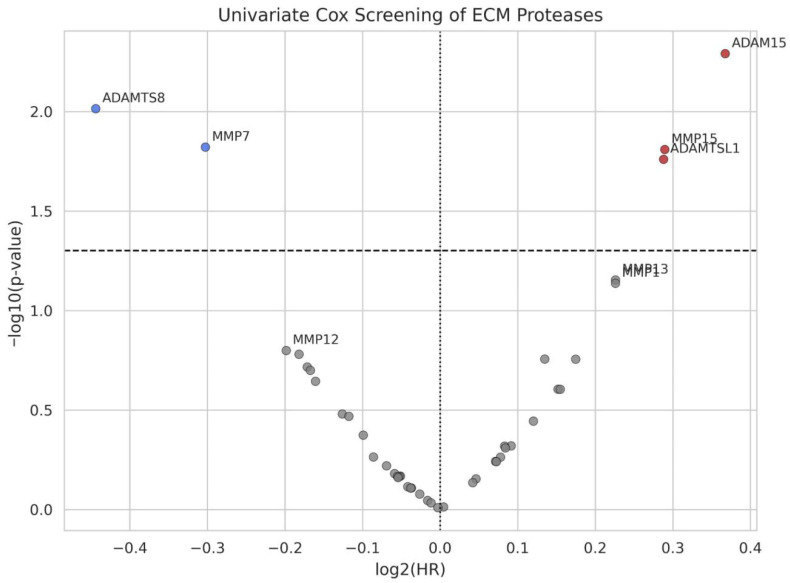
Volcano plot of univariate Cox screening for ECM proteases. Each point represents one protease. The x-axis shows log2(HR); the y-axis shows –log10(*p*-value). The dashed horizontal line marks *p* = 0.05. Statistically significant risk factors (red) and protective factors (blue) are labelled.

**Figure 2:**
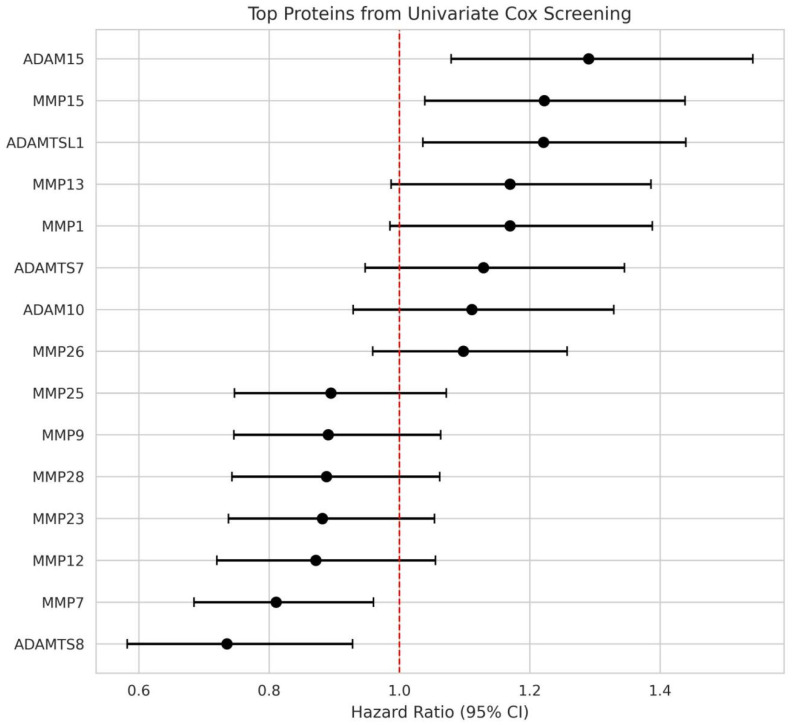
Forest plot of top-ranked proteases from univariate Cox screening. Hazard ratios (HR) with 95% confidence intervals are shown. Points to the right of the dashed red line (HR = 1) indicate increased risk; points to the left indicate a protective association.

In the full cohort (Table 2), five protease features crossed *p* < 0.05 in univariate Cox screening, as visualized in the volcano plot (Figure 1) and forest plot (Figure 2). Several additional proteins showed directionally consistent but non-significant trends, including MMP13 and MMP1 with HR near 1.17 and *p*-values around 0.07.

### 3.2. Subtype-Specific Associations

Subtype analysis revealed distinct prognostic patterns across the molecular groups (Table 3), consistent with the known biological heterogeneity of breast cancer [33]. In the Basal-like subtype, MMP10 was the only protein among the top candidates to reach statistical significance, correlating with increased risk (HR = 1.62, *p* = 0.020), in line with prior evidence for MMP enrichment in aggressive subtypes [19,22]. The limited number of significant associations in this subgroup may be partially attributable to the compositional properties of pTPM quantification: in the proliferation-dominant transcriptomes characteristic of Basal-like/TNBC biology, high expression of cell-cycle and growth-related genes may compress the relative pTPM values of lower-expressed proteases, attenuating their detectable prognostic signal. For the HER2-positive subtype, MMP19 (HR = 0.37, *p* = 0.036) and MMP28 (HR = 0.25, *p* = 0.046) demonstrated protective effects, whereas ADAMTS18 was associated with increased risk (HR = 2.79, *p* = 0.047). Within the Luminal A subtype, several genes were linked to elevated risk, most notably ADAMTS3 (HR = 1.65, *p* = 0.004), alongside ADAMTSL1 (HR = 1.41, *p* = 0.010), MMP26 (HR = 1.18, *p* = 0.020), and ADAM10 (HR = 1.44, *p* = 0.040) [27,37]; conversely, MMP7 conferred a protective effect (HR = 0.65, *p* = 0.013). In the Luminal B subtype, both ADAM22 (HR = 0.65, *p* = 0.038) and MMP25 (HR = 0.63, *p* = 0.042) were identified as protective factors. Finally, in the Normal-like subtype, ADAM15 exhibited a notably large risk effect size (HR = 6.25, *p* = 0.008), with MMP15 (HR = 2.27, *p* = 0.036) and ADAMTS7 (HR = 2.55, *p* = 0.043) also correlating with increased prognostic risk [32].

It should be noted that the Normal-like subtype comprises only 34 patients in this cohort, and the extreme hazard ratio for ADAM15 (95% CI: 1.60–24.46) reflects the statistical instability inherent to small-sample subgroup analyses; this association should therefore be considered hypothesis-generating and requires prospective validation in larger Normal-like cohorts. The appearance of ADAM15 and MMP15 as global risk factors despite their strongest subtype-specific signal arising in Normal-like disease is explained by the cohort composition: the TCGA BRCA dataset is numerically dominated by Luminal A (*n* = 433), and global Cox estimates are weighted accordingly. In the full cohort, ADAM15 reaches global significance (HR = 1.29, *p* = 0.005) because its risk contribution, though strongest in Normal-like tumors, is directionally consistent and detectable at modest effect sizes across other subtypes as well (e.g., HR = 1.70 in HER2-positive, *p* = 0.094; HR = 1.58 in Basal-like, *p* = 0.070; Table A1).

The co-occurrence of risk-associated and protective factors within the same molecular subtype, most notably ADAMTSL1 (HR = 1.41, risk) and MMP7 (HR = 0.65, protective) both reaching significance in Luminal A, reflects biologically distinct and non-redundant roles within the same tumor microenvironment. ADAMTSL1 is a matricellular glycoprotein that modulates TGF-β bioavailability and stromal stiffness, promoting invasive progression [14,15], whereas MMP7 (matrilysin) has been shown to shed and inactivate membrane-bound factors that promote tumor cell survival, and its protective role in luminal breast cancer is consistent with evidence that MMP7-mediated processing of E-cadherin and Fas ligand can restrict cell proliferation and promote apoptosis in hormone receptor-positive disease [32]. The presence of both signals in Luminal A does not represent a contradiction; rather, it reflects the complexity of the ECM protease network, in which individual enzymes operate on distinct substrates and exert opposing net effects on patient survival within the same subtype context [11].

### 3.3. Co-Expression Structure of Extracellular Proteases

**Figure 3:**
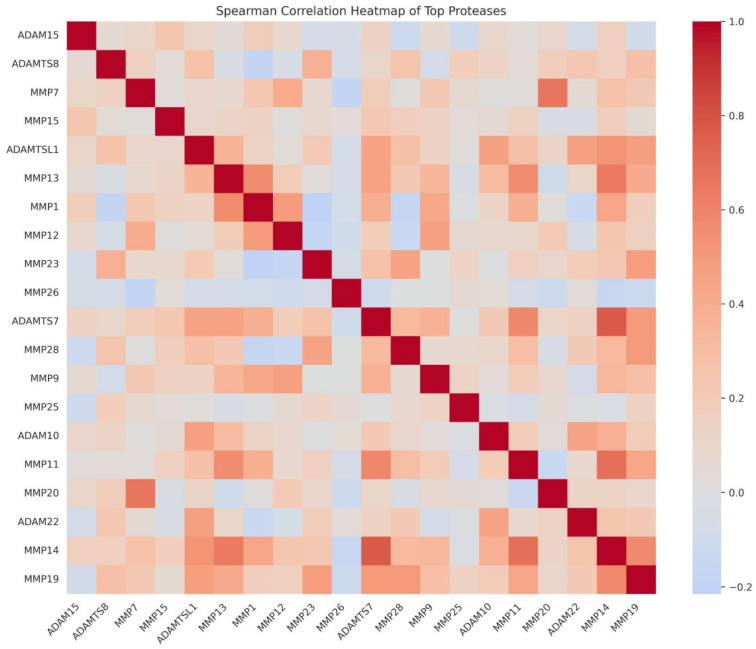
Spearman correlation heatmap of the top ECM proteases across the full cohort. Color intensity reflects the magnitude of pairwise co-expression (red = positive, blue = negative). Most pairs display positive co-expression, with MMP14 emerging as a correlation hub.

**Figure 4:**
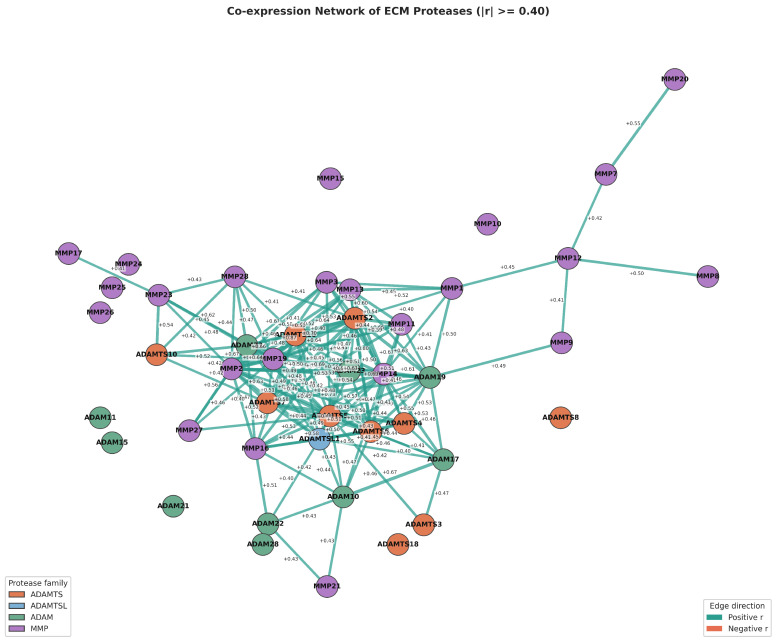
Co-expression network of ECM proteases (|r| ≥ 0.40). Each node represents one protease; edges connect pairs with Spearman |r| ≥ 0.40. A densely connected hub cluster is visible at the centre, reflecting coordinated transcriptional regulation.

To assess functional coupling, we computed pairwise co-expression correlations across the patient cohort and visualized the strongest relationships (Figure 3 and Figure 4). Most pairs showed positive correlations, consistent with coordinated regulation. MMP14 appeared as a key hub, with the strongest correlation to ADAMTS7 (r = 0.73) and further strong links to MMP11 (r = 0.63) and MMP13 (r = 0.59). Negative correlations were weak, for example, MMP1 versus MMP28 (r = −0.13), indicating that the overall pattern reflects co-expression rather than mutual exclusion.

### 3.4. Predictive Modeling and Risk Stratification

**Figure 5:**
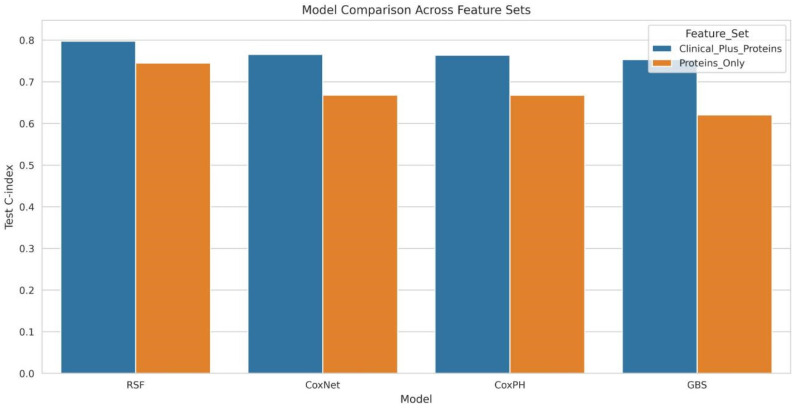
Test C-index comparison across four survival model families (CoxPH, CoxNet, RSF, GBS) and two feature sets (Clinical + Proteins vs. Proteins only). The integrative Random Survival Forest configuration achieves the highest discrimination (C-index = 0.797).

Integrative multivariable models (Clinical + Protease mRNA) consistently outperformed single-modality (mRNA-only) approaches across all tested algorithms. The Random Survival Forest (RSF) achieved the highest overall discrimination, improving from a C-index of 0.745 (mRNA only) to 0.797 (integrated) (Figure 5). Parallel synergistic improvements were observed in Gradient Boosting Survival (0.620 to 0.753), CoxNet (0.668 to 0.765), and Cox Proportional Hazards (0.667 to 0.764).

To quantify how much of the integrative model’s performance is attributable to the ECM protease features specifically, a clinical-only Cox proportional hazards model fitted on age and stage alone using the same 70/30 train-test split achieved a held-out C-index of 0.742 (95% bootstrap CI 0.636–0.826 over 1000 resamples; Table A3). The integrative RSF therefore contributed ΔC = 0.055 of discrimination beyond the clinical-only baseline, reflecting the independent prognostic value of the ECM protease transcript features.

**Figure 6:**
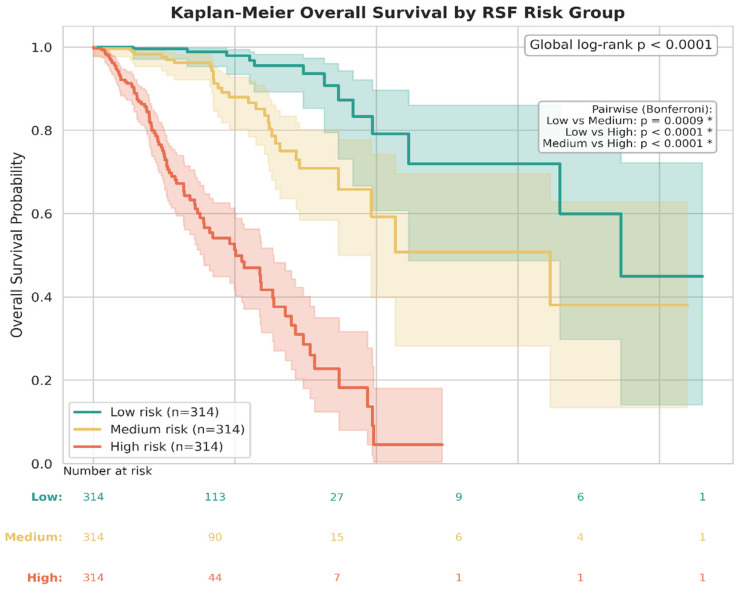
RSF-Based Survival Curves by Risk Group Showing Distinct Clinical Stratification. * shows statistical significance with *p* values as indicated in the inserted upper right panel.

To validate clinical stratification, observed overall survival was compared across the three RSF-derived risk groups (Low, Medium, High) using a multivariate log-rank test. The test confirmed highly significant separation (global log-rank *p* < 0.0001). Pairwise comparisons with Bonferroni correction demonstrated that all three group contrasts were independently significant: Low vs. Medium (*p* = 0.0009), Low vs. High (*p* < 0.0001), and Medium vs. High (*p* < 0.0001). The corresponding Kaplan–Meier observed survival curves with 95% confidence intervals and number-at-risk tables are shown in Figure 6. These results confirm that the RSF-derived continuous risk score generates three clinically and statistically distinguishable prognostic strata, not merely nominal groupings.

The clinical validity of this stratification was confirmed by event counts: the Low-Risk group (*n* = 314) recorded only 13 mortality events, whereas the High-Risk group (*n* = 314) recorded 93 events (Table 4). Five-fold cross-validation of the RSF model confirmed stability (mean C-index 0.735, SD 0.051; Table 5).

### 3.5. Patient-Level Validation

To assess prediction stability relative to biomarker selection, reduced input versions (Top 10, Top 5, and Significant-only) were compared against complete profiles across subtypes:

- Basal & HER2: Full-profile predictions yielded the lowest risk scores. Reduced profiles shifted the survival curve downward but remained tightly grouped and consistent, maintaining trajectory stability even when approaching the 50% survival mark.
- Luminal B & Normal-like: Reduced sets predicted lower survival than the full profile but were internally consistent, showing only modest separation. In the Normal-like example, at 3 years, full-profile survival was 94.4%, compared to 79.4% (Top 10) and 71.6% (Significant-only).
- Luminal A: Exhibited the largest gap between the full profile (Risk Score 18.22) and reduced profiles (Risk Scores 43.12–47.33), highlighting that broader biomarker integration is highly sensitive in this specific subtype.

### 3.6. Stage and Age Effects (Non-Monotonic Predictions)

Patient-level simulations revealed that survival does not strictly follow clinical tumor stage, demonstrating that the model successfully overrides monotonic clinical assumptions:

- Basal-like: A Stage I patient (83 years old) exhibited the highest risk score (30.37) and poorest survival, while a younger Stage IIA patient had the most favorable outcome.
- HER2-positive: The Stage I case presented the poorest outlook (risk score 40.10) compared to a Stage IIIA case (risk score 20.62). This indicates that the specific protease profile drives high-risk predictions independent of standard anatomical staging.
- Luminal A: An advanced Stage IV case (35 years old) showed the poorest outcome, demonstrating that despite young age, the combined biomarker profile and stage heavily dominated the survival shift.

### 3.7. External Validation in the METABRIC Cohort

The locked RSF model, trained exclusively on TCGA-BRCA, was applied to the METABRIC cohort as an independent external validation (Table 6). After matching expression profiles to clinical survival data, 1979 METABRIC patients were available for evaluation (events: *n* = 1143; censored: *n* = 836; median follow-up: approximately 116.5 months). The model achieved an external C-index of 0.581 (95% CI 0.562–0.598), indicating modest but statistically significant discrimination of survival in an independent cohort from a different institution and assayed on a different expression platform (Illumina microarray vs. RNA-Seq). Stratification of METABRIC patients into Low, Medium, and High risk groups using thresholds derived from the training cohort yielded statistically significant separation of overall survival (log-rank *p* < 0.0001), indicating that the directional prognostic information of the ECM protease signature partially transfers to the independent METABRIC cohort, although the absolute discriminative accuracy is modest and clearly below internal held-out test performance (ΔC ≈ 0.22 versus the internal RSF C-index of 0.797). Across the three risk strata, observed event rates showed a clear monotonic gradient (Low: 240/529 events, 45.4%; Medium: 333/612 events, 54.4%; High: 570/838 events, 68.0%; multivariate log-rank χ^2^ = 74.35, *p* < 0.0001), supporting preserved risk ordering despite the reduced absolute concordance.

## 4. Discussion

The continuous survival analysis identified the core biological drivers of breast cancer prognosis. ADAM15 was flagged as the strongest global risk factor (HR = 1.29), consistent with its established role in ectodomain shedding and tumor dissemination. Subtype-specific markers such as MMP10 in Basal-like and ADAMTS3 in Luminal A tumors were identified. Internally, the analysis showed that integrating molecular protease data with clinical factors improved discrimination relative to transcript-only models across all tested model families. However, the independent METABRIC validation requires a cautious interpretation: the model retained statistically significant risk-group separation, but individual-level discrimination was modest, indicating that the signature is not yet clinically deployable without additional validation and calibration.

The continuous RSF model excels at handling collinearity, such as the heavily correlated MMP14 network, without information loss, generating a highly personalized survival trajectory over a five-year timeline. To contextualize model performance against an honest clinical benchmark, we fitted a clinical-only Cox proportional hazards model using age and ordinal stage alone on the same cohort and the same 70/30 train-test split (Table A3). This baseline achieved a held-out C-index of 0.742 (95% bootstrap CI 0.636–0.826), which is in the range reported in the literature for staging-based prognostic tools such as the Nottingham Prognostic Index and Adjuvant! Online. The integrative RSF achieved a held-out C-index of 0.797, corresponding to a ΔC-index of 0.055 in favor of the integrated model. The clinical-only baseline is used here as the closest available proxy for NPI and Adjuvant! Online because the tumor size in centimetres, the exact count of positive lymph nodes, and the histologic grade required by the true NPI formula were not present in the harmonized UCSC Xena clinical release accessed for this study; a per-patient computation of Oncotype DX was similarly not feasible because our transcriptomic feature set is restricted to the MMP, ADAM and ADAMTS families and does not cover the 21-gene Recurrence Score panel. A direct head-to-head benchmark against NPI, Adjuvant! Online, and Oncotype DX on an independent cohort uniformly annotated for tumor size, nodal burden, histologic grade, and the Oncotype gene panel therefore remains an important direction for prospective validation [2,3,5,6].

The reduction from internal held-out performance (C-index = 0.797) and cross-validation performance (mean C-index = 0.735) to external METABRIC performance (C-index = 0.581) is biologically and methodologically plausible. Several factors may contribute platform differences between TCGA RNA-Seq pTPM and METABRIC Illumina microarray expression data, residual batch effects despite independent scaling, differences in cohort composition and treatment era, mismatch in available clinical variables and stage annotation, the use of all-cause overall survival rather than breast cancer-specific survival, and biological heterogeneity across molecular subtypes. Although no final protease feature was missing after alias mapping, expression-scale differences can still attenuate model transportability. The external result also indicates some overfitting or cohort-specific learning by the RSF, despite internal cross-validation, and therefore supports presenting the model as a candidate prognostic framework rather than a validated clinical decision tool.

In the global cohort, ADAM15, MMP15, and ADAMTSL1 demonstrated significant risk associations. The strong risk signal from ADAM15 (HR = 1.29, *p* = 0.005) aligns with its established role in ectodomain shedding and integrin-mediated tumor dissemination [12,13]. MMP15 (MT2-MMP), a membrane-type matrix metalloproteinase that activates pro-MMP-2 at the cell surface and processes pericellular substrates to promote invasive microenvironment remodeling, is biologically consistent with the risk direction observed here; its strongest subtype-specific signal arose in Normal-like tumors (HR = 2.27, *p* = 0.036; Table 3), with a directionally concordant effect at the full-cohort level (HR = 1.22, *p* = 0.016; Table 2) [9,10]. It is important to clarify that these proteins function as global risk factors in the statistical sense, they are associated with increased mortality risk relative to the full TCGA BRCA cohort, regardless of which molecular subtype contributes the strongest individual signal. A protein can reach global significance through a consistent directional effect across multiple subtypes even if its strongest individual association arises in a numerically smaller subgroup. The designation of a protein as a “global risk factor” therefore reflects its cohort-level prognostic weight, not an assertion that it is exclusively or even primarily active in one subtype. The protective effects of ADAMTS8 and MMP7 at the global level are consistent with their roles as tumor microenvironment regulators [32], and MMP7’s protective effect in Luminal A specifically is consistent with its known role in processing membrane-bound apoptotic ligands in hormone receptor-positive disease. ADAMTS8, often referred to as METH2, has been reported to exert anti-angiogenic activity through suppression of VEGF-driven endothelial signaling and to be downregulated across several epithelial tumors; its protective association with survival in the present cohort is consistent with its proposed role as a stromal anti-angiogenic factor whose loss is permissive for vascular remodeling during tumor progression [14,15,32].

### Limitations

The foremost limitation of this study is the restricted dataset size, particularly the small sample size within the Normal-like subgroup, which constrains the training capacity of complex algorithms. Subtype-specific analyses in small subgroups, especially Normal-like (*n* = 34) and HER2-positive (*n* = 61), are statistically underpowered, and extreme hazard ratio estimates in these groups (e.g., ADAM15, HR = 6.25 in Normal-like; 95% CI: 1.60–24.46) should therefore be interpreted as exploratory and hypothesis-generating rather than confirmatory. These associations require validation in larger, subtype-stratified prospective cohorts before any clinical conclusions can be drawn.

A second fundamental limitation is the reliance on transcript abundance (pTPM) rather than direct protein activity measures. Because protease function is strictly regulated by post-translational activation, zymogen cleavage, and inhibition by endogenous inhibitors such as TIMPs, mRNA levels serve only as a proxy for functional proteolytic activity [6,7]. Genome-wide studies of mRNA–protein correlation across human tissues and cancer types report median Spearman correlation coefficients in the range of approximately 0.40–0.60, with substantial variability across individual proteins [7]. For metalloprotease family members specifically, post-translational regulation is particularly pronounced, as many MMPs and ADAMs are secreted as inactive zymogens that require extracellular activation. Transcript abundance may therefore systematically overestimate functional enzyme activity in the tumor microenvironment. These considerations underscore the importance of future validation using proteomic or activity-based protease profiling approaches.

A third limitation relates to the compositional nature of pTPM quantification. Because all protein-coding gene abundances are constrained to sum to one million per sample, TPM values are mathematically interdependent across genes. In Basal-like tumors, which are characterized by extremely high expression of proliferation-related genes, this compositional constraint may compress the relative pTPM values of lower-expressed proteases, attenuating their detectable prognostic signal and potentially contributing to the limited number of significant associations observed in this subtype (Table 3). The pTPM matrices used in this study were accessed through the UCSC Xena platform as pre-processed outputs of the TOIL recompute pipeline; raw STAR integer count matrices with the subtype-level clinical annotations required for this analysis were not available. Consequently, DESeq2 Variance Stabilizing Transformation (VST), which requires non-negative integer counts and cannot be applied to continuous pre-normalized values such as pTPM, was not feasible within the scope of the current study. Future work using raw STAR count data is therefore encouraged, particularly to test whether VST-based normalization strengthens the protease prognostic signal in the Basal-like subtype.

A fourth limitation concerns the coverage of the protease panel analyzed. Three enzymes prominently discussed in the Introduction—ADAM8, ADAM9, and ADAM12—were not present as protein-coding transcripts in the harmonized UCSC Xena pTPM matrix used here and therefore could not be formally tested, although their reported relevance to breast cancer biology is acknowledged. ADAM28 was present as a column in the source file but carried no measurable expression (all missing values) and was automatically excluded during preprocessing. Literature-established MMPs such as MMP1, MMP9, and MMP11 were included in the analysis: MMP1 showed a directionally consistent but non-significant risk trend in the full cohort (HR = 1.17, *p* = 0.073), whereas MMP9 and MMP11 did not reach significance at either the cohort or subtype level (Table 2 and Table A1). These findings do not contradict the published literature, but likely reflect the combined effects of transcript-level quantification, compositional pTPM constraints, and limited sample size within subtypes. Relatedly, a direct head-to-head benchmark of the integrative RSF against the exact Nottingham Prognostic Index and the Oncotype DX Recurrence Score could not be performed on this cohort, because the harmonized UCSC Xena clinical release does not contain the three variables required by the NPI formula (tumor size in centimetres, exact positive lymph node count, and histologic grade) and our transcriptomic feature set does not cover the 21-gene Oncotype panel; the age-plus-stage clinical Cox model reported in the Discussion (Table A3, C-index = 0.742, 95% CI 0.636–0.826) is the closest available honest proxy.

A fifth limitation concerns the use of overall survival (OS) as the primary endpoint. Although OS is an objective and universally available outcome measure, it captures all-cause mortality rather than breast cancer-specific mortality. In an older patient population such as the TCGA BRCA cohort, which includes patients up to 90 years of age, deaths from competing causes unrelated to breast cancer biology may contribute substantially to the observed signal. This is particularly relevant to the patient-level simulation examples presented in Section 3.6, where an 83-year-old Stage I patient exhibited the highest predicted risk score; in this case, the poor predicted outcome may reflect competing-cause mortality associated with advanced age rather than aggressive ECM protease biology per se. Breast cancer-specific survival (BCSS) or disease-free survival (DFS) would provide more precise endpoints for evaluating protease-driven tumor biology, but cause-of-death granularity and recurrence data were not consistently available with sufficient completeness in the TCGA BRCA dataset used here.

An additional limitation is the modest external discriminative performance in METABRIC. Although risk-group separation was statistically significant, the external C-index of 0.581 indicates limited individual-level ranking accuracy. The significant log-rank result is strengthened by the large external sample size and should not be overinterpreted as proof of clinical utility. This performance drop may reflect cross-platform expression differences, residual batch effects, endpoint and treatment-era differences, incomplete harmonization of clinical variables, and cohort-specific model fitting. Future work should therefore include platform-harmonized preprocessing, calibration assessment, time-dependent performance metrics, decision-curve analysis, and prospective validation in cohorts with uniformly annotated treatment, recurrence, and breast cancer-specific survival endpoints.

Finally, the TCGA BRCA cohort is clinically heterogeneous with respect to both tumor biology and treatment exposure. Samples were collected between approximately 1989 and 2013, spanning major shifts in standard-of-care management, including the introduction of taxane-based chemotherapy, aromatase inhibitors, and HER2-targeted therapy (trastuzumab). Treatment heterogeneity across this period may therefore introduce systematic confounding into survival estimates that cannot be fully controlled without detailed individual treatment data, which were not uniformly available in the TCGA clinical annotation.

## 5. Conclusions

This study shows that integrating extracellular matrix protease expression profiles, specifically the MMP, ADAM, and ADAMTS families, with standard clinical covariates improves internal breast cancer survival prediction in the TCGA-BRCA cohort (ΔC-index = 0.055 over an age-plus-stage clinical Cox baseline; Table A3). External validation in METABRIC provided partial support for transportability, with statistically significant risk-group separation but modest individual-level discrimination (C-index = 0.581, 95% CI 0.562–0.598). Therefore, the identified proteases should be interpreted as candidate prognostic markers associated with risk stratification rather than definitively established clinical predictors.

The central contribution of this work is the integration of ECM protease transcript data with standard clinical variables within continuous machine learning survival models. The Random Survival Forest captured non-linear and potentially subtype-dependent interactions and generated personalized relative-risk trajectories. However, the external validation results indicate that these trajectories require further calibration and validation before they can be used for individual clinical decision-making.

Overall, this integrative framework provides a biologically motivated foundation for future targeted multiplex assays, but its current role should remain exploratory. Validation in independent, prospectively collected, platform-harmonized cohorts with breast cancer-specific endpoints is the critical next step before ECM protease signatures can be incorporated into precision oncology workflows.

## Figures and Tables

**Table 1:** Cohort demographic and clinical characteristics.

Characteristic	Category	*n*	%
Total patients	—	942	100%
Sex	Female	942	100%
Age at diagnosis	Median (range)	58 years (26–90)	—
Vital status	Deceased (event)	135	14.3%
	Alive/censored	807	85.7%
Molecular subtype	Luminal A	433	46.0%
	Luminal B	156	16.6%
	Basal-like	155	16.5%
	HER2-positive	61	6.5%
	Normal-like	34	3.6%
	Unknown/not assigned	103	10.9%
Clinical stage	Stage I	170	18.0%
	Stage II	529	56.2%
	Stage III	212	22.5%
	Stage IV	16	1.7%
	Missing/unparseable	15	1.6%

**Table 2:** Prognostic Associations of Candidate Proteins in the Full Study Cohort.

Protein	Concordance	HR	*p*-Value
ADAM15	0.614	1.290	0.005
ADAMTS8	0.629	0.735	0.010
MMP7	0.556	0.811	0.015
MMP15	0.576	1.222	0.016
ADAMTSL1	0.523	1.221	0.017
MMP13	0.519	1.170	0.070
MMP1	0.544	1.170	0.073
MMP12	0.535	0.872	0.159
MMP23	0.550	0.882	0.166
MMP26	0.496	1.098	0.176
ADAMTS7	0.539	1.129	0.176
MMP28	0.559	0.888	0.192
MMP9	0.505	0.891	0.200
MMP25	0.539	0.895	0.227

**Table 3:** Subtype-Specific Prognostic Associations in Breast Cancer.

Breast Cancer Subtype	*n*	Protein	95% CI	HR	*p*-Value
Basal-like	155	MMP10	1.08–2.43	1.62	0.020
HER2-positive	61	MMP19	0.15–0.94	0.37	0.036
		MMP28	0.06–0.97	0.25	0.046
		ADAMTS18	1.01–7.66	2.79	0.047
Luminal A	433	ADAMTS3	1.17–2.34	1.65	0.004
		ADAMTSL1	1.09–1.84	1.41	0.010
		MMP7	0.46–0.92	0.65	0.013
		MMP26	1.03–1.36	1.18	0.020
		ADAM10	1.02–2.04	1.44	0.040
Luminal B	156	ADAM22	0.43–0.98	0.65	0.038
		MMP25	0.41–0.98	0.63	0.042
Normal-like	34	ADAM15	1.60–24.46	6.25	0.008
		MMP15	1.05–4.91	2.27	0.036
		ADAMTS7	1.03–6.33	2.55	0.043

**Table 4:** RSF Risk Group Summary Statistics.

Risk Group	*n* Patient	*n* Events	Event Rate	Median Survival (Days)
Low	314	13	4.1%	1164
Medium	314	29	9.2%	955
High	314	93	29.6%	665

*n* Events and Event Rate are based on the test set. Median Survival is reported in days from diagnosis to last follow-up or death.

**Table 5:** Five-Fold Cross-Validation C-Index of the Final Integrative RSF Model.

Fold	Train C-Index	Test C-Index
1	0.905	0.715
2	0.909	0.673
3	0.897	0.811
4	0.896	0.755
5	0.896	0.720
Mean ± SD	0.901 ± 0.006	0.735 ± 0.050

Train and test C-index values across five stratified folds. The training C-index is consistently high (~0.90), while test performance (mean 0.735) reflects realistic generalisation capacity.

**Table 6:** Independent external validation of the locked integrative RSF model in METABRIC.

Cohort	METABRIC (External)
*n*_Patients	1979
*n*_Events	1143
*n*_Censored	836
*n*_Features_Used	17
Missing_Protease_Genes	None
External_C_Index	0.5805

## Data Availability

The data presented in this study are derived from the following resources available in the public domain: (1) The Cancer Genome Atlas (TCGA) Breast Invasive Carcinoma (BRCA) dataset, available at https://portal.gdc.cancer.gov; and (2) harmonized transcriptomic and clinical data accessed via the UCSC Xena platform, available at https://xenabrowser.net (accessed on 1 March 2026). The full analysis code supporting this study is publicly available at the repository listed in Appendix C.

## Abbreviations

The following abbreviations are used in this manuscript:

ADAM	A Disintegrin and Metalloproteinase
ADAMTS	A Disintegrin and Metalloproteinase with Thrombospondin Motifs
C-index	Concordance Index
ECM	Extracellular Matrix
GBS	Gradient Boosting Survival
HR	Hazard Ratio
MMP	Matrix Metalloproteinase
NPI	Nottingham Prognostic Index
pTPM	Protein-coding Transcripts Per Million
RSF	Random Survival Forest
TCGA	The Cancer Genome Atlas
TNBC	Triple-Negative Breast Cancer

## Appendix A. Subtype-Specific Cox Proportional Hazards Results

**Table A1:** Subtype-specific Cox proportional hazards regression results for all ADAM/ADAMTS/MMP proteins across BRCA molecular subtypes. HR: hazard ratio; CI: confidence interval; C-Index: concordance index. Significant associations (*p* < 0.05) are indicated.

Subtype	*N*	Protein	HR	HR Lower 95% CI	HR Upper 95% CI	*p*-Value	C-Index	Significant
BRCA_LumA	433	ADAMTS3	1.654	1.172	2.336	0.0042	0.582	Yes
		ADAMTSL1	1.413	1.087	1.836	0.0097	0.582	Yes
		MMP7	0.651	0.464	0.915	0.0134	0.682	Yes
		MMP26	1.181	1.027	1.359	0.0197	0.513	Yes
		ADAM10	1.440	1.017	2.039	0.0396	0.568	Yes
		ADAMTS8	0.754	0.542	1.049	0.0941	0.670	No
		MMP12	0.640	0.380	1.080	0.0943	0.600	No
		MMP21	1.243	0.936	1.651	0.1327	0.575	No
		MMP10	0.780	0.562	1.083	0.1377	0.587	No
		MMP15	1.269	0.905	1.779	0.1671	0.611	No
		ADAMTS18	1.208	0.903	1.616	0.2034	0.477	No
		MMP24	1.163	0.905	1.494	0.2378	0.503	No
		ADAM17	1.194	0.876	1.629	0.2617	0.508	No
		ADAM21	0.820	0.575	1.170	0.2731	0.519	No
		ADAMTS6	1.156	0.878	1.522	0.3027	0.485	No
		MMP11	0.852	0.614	1.182	0.3365	0.593	No
		ADAMTS7	1.140	0.831	1.564	0.4161	0.492	No
		MMP19	0.904	0.690	1.183	0.4607	0.573	No
		MMP9	0.903	0.672	1.213	0.4985	0.515	No
		MMP27	1.102	0.823	1.475	0.5157	0.443	No
		MMP23	0.911	0.681	1.218	0.5289	0.570	No
		ADAMTS16	0.918	0.683	1.235	0.5728	0.616	No
		MMP20	0.728	0.204	2.601	0.6247	0.560	No
		MMP13	0.928	0.681	1.265	0.6360	0.549	No
		ADAM22	1.071	0.804	1.428	0.6380	0.480	No
		MMP8	0.914	0.621	1.344	0.6461	0.494	No
		ADAMTS5	1.066	0.806	1.409	0.6551	0.471	No
		MMP16	1.067	0.791	1.439	0.6714	0.432	No
		MMP28	0.941	0.674	1.314	0.7208	0.577	No
		ADAM19	0.952	0.702	1.291	0.7517	0.534	No
		MMP1	1.047	0.738	1.485	0.7987	0.474	No
		MMP3	0.964	0.713	1.304	0.8136	0.605	No
		ADAM23	0.965	0.715	1.302	0.8175	0.568	No
		ADAM11	0.952	0.619	1.465	0.8243	0.536	No
		ADAMTS2	1.031	0.771	1.378	0.8356	0.456	No
		ADAMTS10	0.976	0.716	1.331	0.8795	0.533	No
		MMP2	0.977	0.718	1.329	0.8799	0.568	No
		ADAMTS4	0.985	0.724	1.341	0.9255	0.521	No
		ADAM15	0.987	0.720	1.353	0.9348	0.466	No
		ADAM33	1.011	0.744	1.373	0.9456	0.429	No
		MMP14	1.007	0.759	1.335	0.9631	0.423	No
		MMP25	0.996	0.755	1.313	0.9752	0.496	No
		MMP17	0.998	0.710	1.402	0.9890	0.483	No
BRCA_LumB	156	ADAM22	0.647	0.429	0.975	0.0376	0.642	Yes
		MMP25	0.635	0.409	0.985	0.0424	0.659	Yes
		ADAMTS8	0.264	0.062	1.129	0.0724	0.600	No
		MMP13	1.376	0.966	1.961	0.0770	0.636	No
		MMP1	1.403	0.959	2.052	0.0808	0.683	No
		MMP28	0.646	0.384	1.086	0.0994	0.595	No
		ADAM23	0.661	0.394	1.109	0.1171	0.567	No
		ADAMTS16	1.361	0.907	2.043	0.1371	0.612	No
		MMP21	0.670	0.387	1.159	0.1519	0.604	No
		ADAMTS3	1.534	0.808	2.916	0.1911	0.560	No
		MMP7	0.724	0.441	1.189	0.2018	0.521	No
		ADAMTS18	0.786	0.540	1.146	0.2108	0.593	No
		MMP2	1.328	0.840	2.101	0.2251	0.660	No
		ADAM19	0.783	0.514	1.192	0.2538	0.501	No
		ADAMTS4	0.793	0.531	1.184	0.2563	0.566	No
		ADAMTS7	1.250	0.813	1.922	0.3093	0.566	No
		ADAM33	1.298	0.782	2.152	0.3128	0.616	No
		ADAM15	1.168	0.841	1.623	0.3547	0.576	No
		MMP11	1.237	0.784	1.951	0.3598	0.590	No
		MMP9	0.829	0.543	1.266	0.3863	0.548	No
		MMP24	1.102	0.878	1.382	0.4027	0.505	No
		ADAMTS10	0.782	0.430	1.423	0.4208	0.569	No
		MMP14	1.195	0.769	1.857	0.4289	0.601	No
		MMP10	1.123	0.839	1.502	0.4358	0.553	No
		ADAMTS6	0.832	0.515	1.343	0.4511	0.464	No
		ADAM21	1.117	0.820	1.522	0.4838	0.565	No
		ADAMTSL1	0.865	0.548	1.365	0.5330	0.536	No
		MMP26	1.199	0.657	2.186	0.5543	0.476	No
		MMP17	0.897	0.623	1.291	0.5597	0.510	No
		ADAMTS2	1.107	0.729	1.683	0.6329	0.599	No
		MMP19	0.905	0.588	1.393	0.6498	0.437	No
		MMP16	1.092	0.719	1.658	0.6810	0.527	No
		MMP23	0.897	0.501	1.608	0.7162	0.544	No
		MMP20	0.562	0.013	24.589	0.7650	0.502	No
		ADAM10	0.950	0.644	1.402	0.7954	0.418	No
		ADAM11	0.938	0.567	1.554	0.8049	0.551	No
		ADAMTS5	1.047	0.669	1.639	0.8409	0.565	No
		ADAM17	1.045	0.677	1.612	0.8422	0.577	No
		MMP27	1.053	0.539	2.060	0.8796	0.571	No
		MMP15	0.975	0.686	1.385	0.8858	0.539	No
		MMP8	0.974	0.675	1.405	0.8868	0.443	No
		MMP3	1.024	0.683	1.535	0.9099	0.572	No
		MMP12	1.020	0.662	1.571	0.9276	0.469	No
BRCA_Basal	155	MMP10	1.619	1.079	2.427	0.0198	0.558	Yes
		ADAM21	1.361	0.997	1.859	0.0523	0.553	No
		ADAM15	1.581	0.963	2.594	0.0702	0.616	No
		MMP8	1.280	0.950	1.725	0.1040	0.559	No
		ADAMTS6	1.430	0.876	2.333	0.1524	0.525	No
		ADAMTS10	1.273	0.869	1.864	0.2159	0.598	No
		ADAMTS3	0.819	0.594	1.129	0.2230	0.530	No
		MMP9	0.735	0.440	1.230	0.2414	0.564	No
		MMP15	1.204	0.875	1.655	0.2543	0.506	No
		ADAMTS8	0.646	0.208	2.002	0.4489	0.563	No
		ADAM23	1.171	0.769	1.784	0.4615	0.493	No
		MMP7	1.197	0.736	1.946	0.4678	0.556	No
		MMP28	1.170	0.760	1.802	0.4760	0.519	No
		MMP1	0.867	0.560	1.341	0.5204	0.586	No
		ADAMTS7	0.885	0.597	1.311	0.5416	0.505	No
		MMP25	1.151	0.729	1.817	0.5463	0.558	No
		ADAMTS16	0.866	0.536	1.398	0.5555	0.507	No
		ADAM11	1.096	0.802	1.499	0.5642	0.466	No
		MMP19	1.156	0.687	1.947	0.5846	0.540	No
		ADAMTS2	1.126	0.715	1.773	0.6079	0.544	No
		MMP17	1.157	0.594	2.254	0.6673	0.554	No
		MMP23	1.146	0.610	2.153	0.6716	0.513	No
		ADAMTS5	1.078	0.740	1.570	0.6950	0.550	No
		ADAM17	0.932	0.640	1.358	0.7142	0.508	No
		ADAMTS4	0.919	0.584	1.447	0.7151	0.571	No
		ADAM10	0.925	0.600	1.426	0.7229	0.528	No
		MMP16	1.072	0.726	1.583	0.7256	0.515	No
		MMP27	1.040	0.796	1.359	0.7731	0.518	No
		ADAM22	0.943	0.610	1.456	0.7898	0.567	No
		ADAMTSL1	1.064	0.667	1.699	0.7946	0.504	No
		MMP24	0.943	0.565	1.573	0.8210	0.562	No
		MMP20	1.021	0.834	1.251	0.8395	0.487	No
		MMP14	0.957	0.571	1.606	0.8686	0.475	No
		MMP12	1.023	0.698	1.501	0.9067	0.515	No
		ADAM19	0.980	0.646	1.486	0.9229	0.514	No
		MMP11	0.978	0.610	1.568	0.9250	0.502	No
		ADAM33	1.032	0.520	2.048	0.9292	0.481	No
		MMP2	0.982	0.635	1.518	0.9344	0.489	No
		MMP21	0.977	0.530	1.800	0.9408	0.529	No
		ADAMTS18	0.980	0.542	1.771	0.9464	0.534	No
		MMP3	0.989	0.666	1.469	0.9551	0.535	No
		MMP13	0.992	0.612	1.611	0.9756	0.526	No
		MMP26	0.000	N/A	N/A	N/A	0.478	No
BRCA_Her2	61	MMP19	0.373	0.149	0.936	0.0357	0.714	Yes
		MMP28	0.249	0.063	0.974	0.0457	0.673	Yes
		ADAMTS18	2.787	1.014	7.659	0.0468	0.625	Yes
		MMP23	0.305	0.082	1.130	0.0755	0.599	No
		ADAM15	1.699	0.913	3.159	0.0942	0.702	No
		MMP24	1.944	0.784	4.821	0.1512	0.535	No
		MMP25	0.555	0.248	1.243	0.1522	0.687	No
		ADAMTS7	0.584	0.276	1.238	0.1605	0.625	No
		ADAMTS8	0.086	0.003	2.663	0.1614	0.646	No
		MMP2	0.604	0.272	1.341	0.2152	0.599	No
		MMP9	0.632	0.296	1.351	0.2369	0.523	No
		MMP16	1.445	0.770	2.710	0.2516	0.574	No
		MMP21	1.213	0.866	1.698	0.2615	0.581	No
		MMP3	0.704	0.347	1.429	0.3308	0.609	No
		ADAM19	0.707	0.348	1.436	0.3376	0.590	No
		ADAMTS2	0.715	0.348	1.471	0.3625	0.584	No
		ADAMTS10	0.630	0.215	1.848	0.3999	0.567	No
		ADAM33	0.614	0.159	2.370	0.4792	0.482	No
		MMP11	0.760	0.338	1.709	0.5072	0.544	No
		ADAM11	0.701	0.239	2.054	0.5166	0.465	No
		MMP12	0.804	0.379	1.704	0.5688	0.546	No
		ADAM17	1.185	0.600	2.339	0.6249	0.590	No
		ADAM22	1.156	0.644	2.074	0.6271	0.629	No
		ADAM21	0.784	0.285	2.160	0.6377	0.540	No
		MMP8	0.842	0.395	1.795	0.6569	0.495	No
		MMP10	0.866	0.455	1.649	0.6623	0.498	No
		ADAMTS6	0.866	0.422	1.780	0.6963	0.552	No
		MMP15	0.902	0.480	1.695	0.7478	0.482	No
		ADAM23	0.866	0.345	2.171	0.7584	0.462	No
		ADAM10	0.911	0.483	1.719	0.7731	0.561	No
		MMP14	0.924	0.511	1.672	0.7948	0.508	No
		ADAMTS5	1.083	0.498	2.357	0.8406	0.491	No
		ADAMTSL1	1.068	0.557	2.046	0.8432	0.491	No
		ADAMTS3	1.071	0.541	2.119	0.8437	0.587	No
		ADAMTS16	1.083	0.490	2.394	0.8443	0.517	No
		MMP7	0.915	0.364	2.296	0.8492	0.465	No
		ADAMTS4	1.055	0.554	2.008	0.8705	0.515	No
		MMP20	1.786	0.001	4998.601	0.8861	0.506	No
		MMP1	1.034	0.560	1.909	0.9159	0.462	No
		MMP13	1.025	0.509	2.062	0.9450	0.543	No
		MMP17	0.986	0.489	1.991	0.9694	0.464	No
		MMP27	1.026	0.214	4.911	0.9748	0.453	No
		MMP26	0.000	0.000	inf	0.9951	0.530	No
BRCA_Normal	34	ADAM15	6.247	1.595	24.459	0.0085	0.878	Yes
		MMP15	2.275	1.054	4.910	0.0363	0.822	Yes
		ADAMTS7	2.553	1.030	6.326	0.0430	0.694	Yes
		MMP11	2.969	0.958	9.200	0.0592	0.722	No
		MMP1	2.030	0.970	4.251	0.0604	0.700	No
		MMP13	1.682	0.837	3.379	0.1442	0.572	No
		MMP10	1.624	0.836	3.155	0.1524	0.583	No
		MMP14	2.112	0.728	6.123	0.1687	0.556	No
		ADAM11	0.263	0.037	1.858	0.1805	0.606	No
		ADAM19	1.600	0.773	3.312	0.2052	0.561	No
		ADAM10	1.783	0.705	4.511	0.2221	0.556	No
		ADAM22	1.823	0.683	4.866	0.2308	0.550	No
		ADAMTS18	0.316	0.041	2.426	0.2677	0.628	No
		MMP24	0.499	0.134	1.856	0.2998	0.694	No
		MMP25	0.451	0.098	2.078	0.3067	0.617	No
		MMP2	1.589	0.652	3.873	0.3082	0.500	No
		MMP3	1.488	0.675	3.277	0.3244	0.622	No
		ADAMTS2	1.502	0.649	3.475	0.3415	0.494	No
		ADAMTS6	1.463	0.652	3.283	0.3568	0.522	No
		MMP17	0.562	0.153	2.061	0.3848	0.650	No
		ADAMTSL1	1.313	0.654	2.635	0.4443	0.489	No
		ADAMTS3	1.408	0.579	3.428	0.4506	0.544	No
		ADAM23	1.408	0.555	3.576	0.4716	0.561	No
		ADAMTS16	1.410	0.547	3.631	0.4768	0.400	No
		MMP9	0.765	0.334	1.752	0.5268	0.589	No
		ADAM17	1.270	0.579	2.784	0.5512	0.406	No
		ADAMTS10	1.419	0.441	4.568	0.5579	0.583	No
		MMP12	0.672	0.171	2.637	0.5689	0.461	No
		MMP8	0.740	0.240	2.283	0.5998	0.500	No
		MMP27	1.245	0.528	2.936	0.6159	0.517	No
		MMP21	0.811	0.318	2.066	0.6598	0.567	No
		MMP20	0.650	0.091	4.638	0.6673	0.472	No
		ADAM33	0.867	0.371	2.028	0.7422	0.589	No
		MMP16	1.109	0.519	2.366	0.7898	0.394	No
		ADAMTS5	0.911	0.456	1.821	0.7921	0.533	No
		MMP7	1.146	0.297	4.425	0.8438	0.489	No
		MMP28	0.964	0.490	1.895	0.9147	0.450	No
		MMP23	0.960	0.428	2.153	0.9215	0.506	No
		ADAM21	1.074	0.222	5.201	0.9292	0.511	No
		ADAMTS8	0.980	0.546	1.759	0.9465	0.467	No
		MMP19	1.023	0.481	2.178	0.9524	0.456	No
		ADAMTS4	1.016	0.480	2.154	0.9661	0.472	No
		MMP26	1.000	0.000	inf	1.0000	0.500	No

N/A: not applied.

## Appendix B. Model Performance Comparison

**Table A2:** Comparison of survival model performance across feature sets and model types. C-Index values for training and test sets are reported. RSF: Random Survival Forest; CoxNet: penalized Cox regression; CoxPH: standard Cox proportional hazards; GBS: Gradient Boosting Survival.

Feature Set	Model	*N* Features	C-Index (Train)	C-Index (Test)
Clinical_Plus_Proteins	RSF	17	0.8910	0.7974
	CoxNet	17	0.7780	0.7654
	CoxPH	17	0.7777	0.7636
	GBS	17	0.8646	0.7531
Proteins_Only	RSF	15	0.8707	0.7449
	CoxNet	15	0.6706	0.6677
	CoxPH	15	0.6711	0.6675
	GBS	15	0.8145	0.6203

**Table A3:** Bootstrapped 95% confidence intervals computed from 1000 resamples of the held-out test set (*n* ≈ 283). The ‘Age + stage’ row is the closest available proxy for staging-based prognostic tools such as NPI and Adjuvant! Online on this cohort (the integrative RSF contributes ΔC = 0.055 beyond the Age + stage clinical-only baseline).

Baseline Features	*N* Features	C-Index (Test)	95% CI (Low)	95% CI (High)
Age only	1	0.647	0.542	0.740
Stage only (ordinal I–IV)	1	0.675	0.577	0.762
Stage only (fine substage)	1	0.669	0.568	0.771
Age + stage (NPI/Adjuvant! proxy)	2	0.742	0.636	0.826
Age + fine substage	2	0.728	0.627	0.815
Integrative RSF (reference)	17	0.797	—	—

## Appendix C. Code

https://github.com/babasrami/ECM-Protease-Breast-Cancer-Survival-Model (accessed on 30 April 2026).
